# Social values as arguments: similar is convincing

**DOI:** 10.3389/fpsyg.2014.00829

**Published:** 2014-08-07

**Authors:** Gregory R. Maio, Ulrike Hahn, John-Mark Frost, Toon Kuppens, Nadia Rehman, Shanmukh Kamble

**Affiliations:** ^1^School of Psychology, Values in Action Centre, Cardiff UniversityCardiff, UK; ^2^Data Collection Methodology, Office of National StatisticsNewport, UK; ^3^Psychology, University of GroningenGroningen, Netherlands; ^4^Psychology, Karnatak UniversityDharwad, India

**Keywords:** social values, political argument, persuasion, plausibility

## Abstract

Politicians, philosophers, and rhetors engage in co-value argumentation: appealing to one value in order to support another value (e.g., “equality leads to freedom”). Across four experiments in the United Kingdom and India, we found that the psychological relatedness of values affects the persuasiveness of the arguments that bind them. Experiment 1 found that participants were more persuaded by arguments citing values that fulfilled similar motives than by arguments citing opposing values. Experiments 2 and 3 replicated this result using a wider variety of values, while finding that the effect is stronger among people higher in need for cognition and that the effect is mediated by the greater plausibility of co-value arguments that link motivationally compatible values. Experiment 4 extended the effect to real-world arguments taken from political propaganda and replicated the mediating effect of argument plausibility. The findings highlight the importance of value relatedness in argument persuasiveness.

## Introduction

“I will choose *freedom* because I think *freedom* leads to *equality*”

George W. Bush (as cited by Anderson, 1999; Anderson, own italics)

Major political ideologies employ co-value argumentation: they appeal to one value in order to support another value. The above example uses the value of freedom to support equality; intriguingly, the 1847 manifesto of the Communist Party (Wheen, 1999) conversely uses the value of equality to support freedom. These are not isolated cases of co-value argumentation; appealing to one social value to validate another unites people as diverse as Plato, who stated that *equality* leads to *friendship* (Prangle, 1988), and Howard Greenspan (Associated-Press, 1999), who stated that “*Honesty* leads to *success* in life and business” (italics added). Although there are a number of other important ways in which values are embedded within argumentation (e.g., Tetlock et al., 1997; Nelson, 2004), co-value argumentation has yet to receive empirical scrutiny.

Two aspects of co-value argumentation make it interesting to examine. First, as in the examples above, these arguments are often quite simple and unelaborated, hardly ever saying why or how two values are connected; it's taken for granted that the recipient will see the connection. Second, the use of co-value argumentation provides people with a huge advantage: social values are seen as important, they are generally viewed positively (Maio and Olson, 1998), and individuals attempt to behave consistently with their values (Rokeach, 1973; Schwartz, 1992; Verplanken and Holland, 2002). It is therefore easy to see why people frequently use co-value argumentation to advance an argument.

However, it should also be important *which* values are paired in such co-value arguments; not all statements involving values are likely to be equally persuasive. This issue is relevant to an unresolved issue in the study of persuasion (Areni and Lutz, 1988; Maio and Haddock, 2007): in a lot of real-world argumentation, it can be unclear which attributes can be plausibly linked to form a strong argument. From the perspective of argumentation research, co-value argumentation constitutes a form of *consequentialist argument*: “We should endorse X, because it will bring about Y” (e.g., Hahn and Oaksford, 2007; Corner et al., 2011). Such arguments invoke so-called “utility conditionals” (e.g., Thompson et al., 2005; Evans et al., 2008; Bonnefon, 2009), that is, conditionals (*if-then* statements) where the antecedent (*if X*..), the consequent (*then Y…*) or both are associated with positive/negative utilities. As a class, such arguments encompass warnings and threats as well as positive recommendations, and their strength depends crucially on the conditional probability, *P*(X|Y), that is, the extent to which Y really will bring about X (see, e.g., Hahn and Oaksford, 2007, 2012).

Resonant with classic theories of attitude function (Smith et al., 1956; Katz, 1960), the strength of co-value argumentation should depend directly on the specific values highlighted by a message. Moreover, psychological research on values should allow explanation and prediction of which value combinations make strong arguments and which do not. The present research focuses on this issue.

### The psychological organization of values

**Figure 1:**
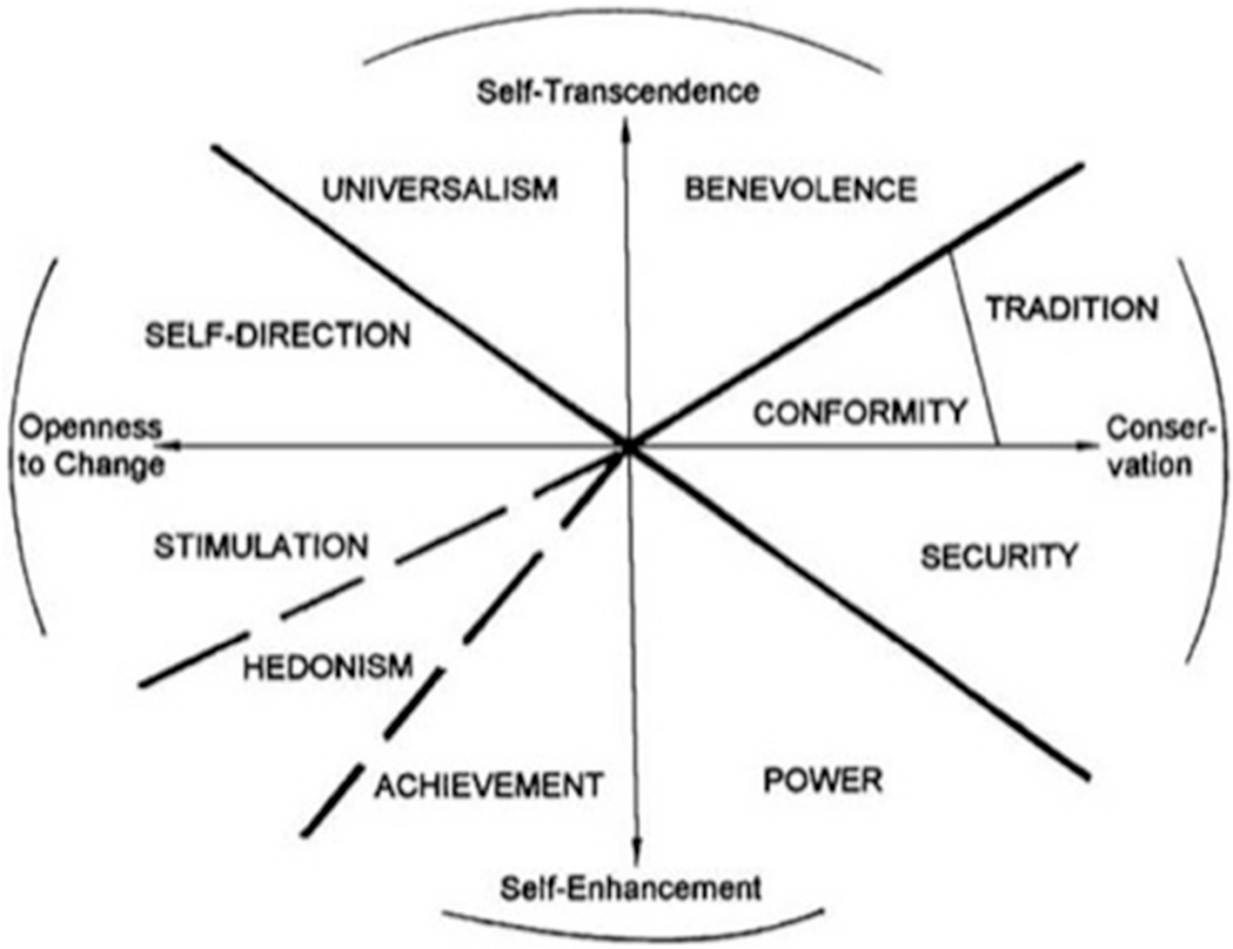
**Schwartz's (1992) model of values**.

This potential role of the specific values can be understood by first considering the psychological organization of values. The most influential and empirically supported model was postulated by Schwartz (1992) and Schwartz and Boehnke (2004). As shown in Figure 1, the crucial feature that creates this model is the type of *motives* that values express. These motives can vary in congruence: actions taken in pursuit of one particular value have psychological, practical, and social consequences that may conflict with the pursuit of another value or be compatible with its pursuit. In the value structure, competing value types are in *opposing* positions around the circle, whereas compatible value types, which fulfill *similar* motives, are in adjacent positions. This structure is supported by patterns of correlations between ratings of the importance of diverse values in over 70 nations (Schwartz, 2006).

Schwartz's (1992) model of social values provides an important theoretical context for the strength of instances of co-value argumentation. The circumplex structure orders values according to motivational congruence. Opposing values are classed as such because the actions taken in their respective pursuit may often conflict in consequence. In other words, pursuing one value is likely to impinge negatively on the pursuit of the other. Conversely, values that fulfill similar motives will be positively correlated in terms of the consequences of actions one might take in their pursuit; and, finally, values that are orthogonal will be more or less independent.

### Implications for co-value arguments

The relative positions along the Schwartz circumplex translate directly into probabilities: the conditional probability *P*(X|Y), that is, the probability that X will obtain given Y, for two values X and Y, will vary systematically depending on their relative location in Schwartz's model. For opposing values, X and Y will be negatively correlated: pursuing X is detrimental to the pursuit of Y, that is *P*(X|Y) will be less than *P*(X), whereas it will be greater than *P*(X) for similar values. Schwartz's model may thus be combined with recent probabilistic approaches to argument quality/argument strength (see, e.g., Hahn and Oaksford, 2007, 2012) to provide principled, a priori predictions about the relative convincingness of different consequentialist arguments combining two values.

Two values should be more plausibly linked when they are motivationally congruent than when they are not; compatibility between congruent values more closely fits personal experience and implicit theories about social values and behaviors. As a result, people should be more persuaded by recommendations based upon co-value argumentation involving similar values than by recommendations based upon co-value argumentation involving dissimilar (unrelated or opposing) values. We call this *the congruence hypothesis*.

Nevertheless, there are two reasons why this possibility is not a foregone conclusion. First, the abstract nature of fundamental values may render them permeable and “fuzzy,” thus people may perceive associations even between opposing values. Indeed, the abstract nature of social values enables politicians, speech writers, business leaders, and others to bring together opposing values and hence appeal to a more diverse spectrum of people (Gordon and Miller, 2004). Thus, people may regard arguments combining diverse values as more compelling because of the sheer breadth of interests that they cover, as noted by observers of political rhetoric (Sanders and Hamilton, 2001). We label this possibility *the breadth hypothesis*.

Second, people may consider other information while making a judgment of persuasiveness. In the context of values, this may include even the association between values in memory. Indeed, there is evidence that motivationally similar and motivationally opposing values are equally strongly associated in memory (Pakizeh et al., 2007; Maio et al., 2009), *independently* of their mere semantic relatedness (Pakizeh et al., 2007). The motivational associations may facilitate the transfer of information between the values and, consequently, increase the strength of any argument that binds them. That is, arguments involving similar values and arguments involving opposing values may *both* be more persuasive than arguments involving unrelated values because the associations in memory between the similar and the opposing values results in greater acceptance of the transfer of information between them. We label this possibility *the association hypothesis*.

### The present research

To test these congruence, breadth, and association hypotheses, we presented participants in our research with arguments that endorsed a target value because it promoted another value (the “reason value”). For example, in Experiment 1, participants in one experimental condition read, “we should encourage helpfulness because it will promote true friendship.” These two values fulfilled similar motives (adjacent in the Schwartz circumplex). We compared participants' ratings of the strength of these arguments with their ratings of the strength of arguments conjoining values that served orthogonal or opposing motives. The results of these comparisons then led to our second experiment, which examined effects of co-value arguments across a wider range of values, while examining the process mediating the obtained effect of value motivations on ratings of argument cogency. The third experiment then tested whether the findings are replicable in a different culture, India, which is more collectivist in orientation than the culture used in the first two experiments (Britain). Finally, the fourth experiment tested whether the results are applicable to understanding longer, more complex co-value arguments.

## Experiment 1

### Method

#### Participants

Participants were 60 undergraduate psychology students (38 women, 14 men, and 8 who did not indicate gender) at a British university, who participated for course credit.

#### Procedure

Participants were informed that they would be taking part in two different “studies” that had been combined because they were short. The experimenter randomly assigned the argument persuasiveness materials and the Schwartz (1992) Value Survey to roles as the “first” or “second” study. After completion of these tasks, participants were debriefed and thanked. During debriefing, no participants reported any suspicions about the purpose of the research and the relatedness of the values.

#### Manipulation of co-value arguments

All participants were presented with an argument for each of three topics. Each argument claimed that encouraging a target value has beneficial effects on behavior that promotes another value (the reason value). The target value and the reason value served either similar, orthogonal, or opposing motives in Schwartz's (1992) value system (see Table 1). Importantly, a pilot study of 21 participants suggested that the pairs of similar, orthogonal, and opposing values did not differ significantly in value importance when considered out of context of the arguments (all *p*s > 0.45).

**Table 1:** **Experiment 1: similar, orthogonal, and opposing “reason values” from the Schwartz (1992) circular model used in the manipulation of arguments**.

**Target value**	**Similar**	**Orthogonal**	**Opposing**
Creativity	Curiosity	Social influence	Social order
Helpfulness	True friendship	Freedom	Success
Self-discipline	Politeness	Broadmindedness	Enjoyment of life

The arguments were almost identical in context and structure in order to reduce any possibility that participants' evaluations of persuasiveness were determined by factors other than the values themselves. For example, one statement cited one of three benefits of creativity:

“Research conducted by the Arts Council has found that increasing people's creativity has beneficial effects. The studies found that encouraging people to be more creative increases their [curiosity in new ideas and methods/ influence and impact on others/ their sense of social order and stability in society].”

As in the examples provided at the beginning of this paper, the statements were all simple in structure, never stating why or how the two values are connected. To reduce task demands that would have arisen from repeated exposure to the same arguments with alternative reason values, we used three target values (creativity, helpfulness, self-discipline) and, in a Latin Square Confounded design (Kirk, 1995), presented participants (in random order) with one argument for each target value, paired with a similar, orthogonal or opposing value, and counterbalanced such that each participant saw one example of each of these value relationships (see Table 1). The order of presentation of the statements was randomized.

#### Measures

***Value importance***. The Schwartz (1992) Value Survey asked participants to rate the importance of 56 social values as a guiding principle in their life. Each value was presented with a standardized definition from the Schwartz Value Survey, and the list included all of the 12 values (both target and reason values) involved in the manipulation of value relatedness (along with the 44 others). The value ratings were then made using a scale from −1 (“opposed to my values”) to 7 (“extremely important”).

***Argument persuasiveness***. After exposure to the arguments, participants were asked to underline the portion of the paragraph that they considered to be the reason for encouraging the target value. This task ensured that participants processed the whole argument (Langer et al., 1978). Next, participants completed four questions to assess the persuasiveness of the arguments: “To what extent do you find this reason persuasive?”, “How convinced were you by the argument that [creativity/ helpfulness/self-discipline] is a good thing?” “To what extent were you convinced that [creativity/ helpfulness/ self-discipline] is good specifically because it increases behavior consistent with [curiosity in new ideas and methods/ influence and impact on others/ their sense of social order and stability in society]?” and “To what extent do you agree with the report's position that [creativity/ helpfulness/self-discipline] is important?”. Participants responded using 10-point scales from 1 (not at all) to 10 (extremely), and responses to these four questions were averaged to create a single measure of persuasion (average α = 0.81). Participants were also asked to list any other factors that made the argument persuasive, but most provided no additional information (and none alluded to conflicts of consequences between values).

### Results and discussion

#### Persuasiveness

**Figure 2:**
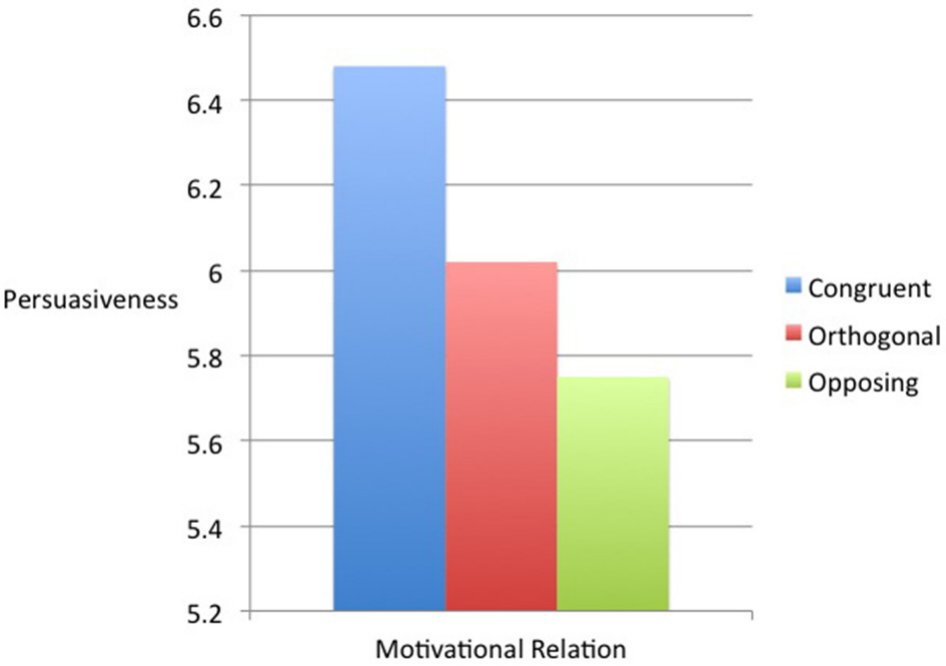
**Persuasiveness as a function of motivational compatibility between values in Experiment 1**.

The Latin Square Confounded technique provided us with participants' argument persuasiveness ratings for each level of value relatedness. These ratings were analyzed using a repeated measures ANOVA, which revealed an effect of value relatedness on participants' ratings of argument persuasiveness, *F*_(2, 118)_ = 4.13, *p* < 0.05. As shown in Figure 2, participants were more persuaded by arguments involving similar value motives (*M* = 6.48) than by arguments involving values that served orthogonal (*M* = 6.02), *t*_(59)_ = 2.01, *p* < 0.05, *d* = 0.52, or opposing motives (*M* = 5.75), *t*_(59)_ = 2.89, *p* < 0.005, *d* = 0.75. There was no significant difference between arguments involving orthogonal and opposing values, *t*_(59)_ = 0.94, *p* < 0.36, *d* = 0.24. Thus, participants found arguments involving similar values more persuasive than arguments involving orthogonal or opposing values.

#### Role of value importance

We also examined participants' mean ratings of the importance of the target and reason values (i.e., creativity and curiosity / true friendship / politeness, in the “similar” condition; helpfulness and social influence / freedom / broadmindedness in the “orthogonal” condition; self-discipline and social order / success / enjoyment of life in the “opposing” condition) using a three-level (similar vs. orthogonal vs. opposing) repeated measures ANOVA. There were no significant differences in value importance across the three types of value relatedness, *F*_(2, 116)_ = 0.84, *p* > 0.4. Therefore, the relatedness effect on argument persuasiveness was not driven by spurious differences in the importance of the values across the value relatedness conditions.

#### Summary

These results provided the first evidence that argument persuasiveness is affected by value relatedness. Of the three hypotheses described in the Introduction, the pattern predicted by the congruence hypothesis was evident in the results. Specifically, participants were more persuaded by arguments involving similar value motives than by arguments involving orthogonal or opposing values. This result indicates that appealing to diverse motives does come at a cost, and it is consistent with extant evidence about the transfer of information between categories of a non-emotional-motivational nature, thereby providing a link between the cognitive research on reasoning and research on persuasion.

## Experiment 2

Given the provocative evidence from Experiment 1, our second experiment examined three additional issues. First, we wished to test whether the previous effects of value relatedness on the rated strength of co-value arguments could be replicated using a design with random pairs of values sampled from around the circular model. Using multilevel modeling, we could also use this method to examine effects of particular value pairs and to test the effects of value relatedness independently of any differences in particular pairs (i.e., the extent to which *any* pair that is highly related shows similar effects).

Second, we tested the hypothesis that similar values may be more persuasive because their linkage is more plausible and fitting with personal experience. One important reason to examine the role of plausibility in consequentialist arguments, such as co-value argumentation, is that consequentialist arguments are influenced *both* by the importance attached to the cited consequence and by the probability that the action will indeed bring about this consequence (see Hahn and Oaksford, 2007, for detailed analysis). Implausible arguments are those where the action is seen to be unlikely to lead to the consequence. Yet, consistent with expectancy-valence perspectives on attitudes and values (Feather, 1995), even plausible arguments, where the action-consequence link is likely, can be unpersuasive if one does not care much about the consequences (e.g., “we should ban cannabis, because cannabis use tends to elicit changes in music preference”). This separation of plausibility and importance is vital because values already inherently convey degrees of importance, and we have shown that their importance is not driving the similarity effect in value co-argumentation. Thus, changes in plausibility are the principal salient alternate path for value linkages to operate.

Finally, we wished to test whether the effect of value relatedness on acceptance of co-value arguments is augmented or diminished by inclinations to think deeply about the arguments. If value relatedness shapes attitudes even when people are carefully scrutinizing value relatedness, it would appear that value relatedness is not simply a default, automatic heuristic, but is an aspect of argument structure that people find useful and rational. For this reason, we included a measure of need for cognition (Cacioppo et al., 1984). This measure taps individual differences in the desire to engage in effortful cognitive activity.

### Method

#### Participants

Participants were 50 undergraduate students (47 women and 3 men) at Cardiff University, who took part for course credit.

#### Procedure

Participants took part individually, and all of the materials were presented using a computer. Participants first rated their agreement with and plausibility of 20 co-value arguments and then completed the 18-item Need for Cognition Scale (Cacioppo et al., 1984). Participants were then debriefed and thanked for their participation.

#### Manipulation of co-value arguments

Examples of the co-value arguments include “Obedience promotes Success” and “National Security promotes Justice.” The computer generated the arguments by randomly selecting pairs of values from a larger set of 24 values. This set included two or three values from each of the 10 motivational domains in Schwartz's (1992) model, with the larger number of values drawn from the larger value domains (e.g., benevolence, self-direction). No participant received the exact same value pairings in their 20 co-value arguments.

We estimated the degrees of relatedness between each value pair that was presented to each participant by manually calculating the angle distance between values in the two dimensional model of individual-level value structure averaged across 20 countries (see Figure 2 in Schwartz, 1992). The estimated angles varied between 0 and 180° (e.g., creativity-honesty = 110°, authority-equality = 180°).

#### Measures

***Agreement and plausibility***. Below each co-value argument, participants were asked, “How much do you agree with this statement?” Participants rated their agreement using a 5-point scale from 1 (strongly disagree) to 5 (strongly agree). After completing this rating, participants were asked, “How plausible did you find the statement?” Plausibility was rated using a 5-point scale from 1 (not at all) to 5 (extremely).

***Need for cognition***. After responding to the co-value arguments, participants completed the 18-item Need for Cognition Scale (Cacioppo et al., 1984). Example items are “I find satisfaction in deliberating hard and for long hours,” “The notion of thinking abstractly is appealing to me,” and “Thinking is not my idea of fun.” Participants responded to each item using a 7-point scale from 1 (strongly disagree) to 7 (strongly agree). We calculated participants' need for cognition as the average score across items, after appropriate reverse-scoring (α = 0.87).

### Results and discussion

We used multilevel modeling to analyse these data. The responses of one particular participant for one particular value pair are the level 1 units. Participants and value pairs are both level 2 units: each response is nested within participants and within value pairs. In all multilevel models the intercept contains a random effect for participants and a random effect for value pairs. The random effect for participants tests whether some participants have higher scores than others (across value pairs). The random effect for value pairs tests whether some value pairs have higher scores than others (across participants). Such a cross-classified structure is appropriate because we wish to generalize our results to all value pairs and all people (see Baayen et al., 2008). Relatedness and need for cognition (*M* = 3.21, *SD* = 0.946) scores were transformed to scores between 1 and 5, as is the case for plausibility, in order to make the regression coefficients more comparable.

We analyzed the extent to which people agreed with each value pair. In Model 1, we added relatedness to the model, and this was a strong predictor of agreement (see Table 2). In Model 2, we added need for cognition and the interaction between relatedness and need for cognition. Need for cognition did not affect agreement, but there was a significant interaction between need for cognition and relatedness. Follow-up simple effects analysis indicated that relatedness was positively related to agreement for people scoring one standard deviation above (*B* = 0.30, *p* < 0.001) and one standard deviation below the mean of need for cognition (*B* = 0.16, *p* < 0.001), but the effect was stronger for people high in need for cognition. Consequently, we tested a model of mediated moderation (see Muller et al., 2005).

**Table 2:** **Experiment 2: multilevel modeling results (standard errors between brackets)**.

	**Model 1**		**Model 2**		**Model 3**	
**FIXED PARAMETERS**						
Intercept	3.24	(0.068)	3.24	(0.068)	3.227	(0.044)
Relatedness	0.230^***^	(0.040)	0.229^***^	(0.040)	0.073^**^	(0.023)
Need for cognition (NFC)			0.066	(0.059)	0.058	(0.044)
Relatedness*NFC			0.073^**^	(0.028)	−0.004	(0.021)
Plausibility					0.753^***^	(0.024)
Plausibility*NFC					0.007	(0.024)
**RANDOM PARAMETERS**						
Participant variance	0.099	(0.031)	0.098	(0.031)	0.056	(0.018)
Value pair variance	0.346	(0.057)	0.347	(0.057)	0.049	(0.019)
Residual variance	0.960	(0.050)	0.954	(0.050)	0.580	(0.030)

*Estimates are unstandardized multilevel regression coefficients. In order to obtain standardized effect sizes, divide the unstandardized coefficients by the square root of the residual variance of Model 1*.

** p < *0.01*;

*** *p < *0.001**.

In Model 3 we added plausibility, the proposed mediator, to the model. Plausibility scores were grand-mean centered. We also added an interaction between need for cognition and plausibility to the model, but did not have specific predictions for this interaction. The plausibility scores were positively related to agreement and this effect was not qualified by need for cognition (see Model 3 in Table 2). Adding plausibility (i.e., the mediator) to the model reduced the effect of relatedness (i.e., the independent variable) and the interaction between relatedness and need for cognition, which is consistent with mediation.

The mediation path that we have not dealt with yet is the effect of relatedness (the independent variable) and its interaction with need for cognition, on plausibility (the mediator). Relatedness affected plausibility (*B* = 0.21, *p* < 0.001), an effect that was qualified by an interaction with need for cognition (*B* = 0.10, *p* < 0.001). Value pairs that are more closely related according to Schwartz' model were indeed judged to be more plausible. However, participants high in need for cognition were more sensitive (i.e., reacted more strongly) to the relatedness of value pairs, compared to people low in need for cognition. Simple slope analysis showed that the relation between relatedness and plausibility is positive and significant for participants one standard deviation above (*B* = 0.30, *p* < 0.001) and one standard deviation below the mean of need for cognition (*B* = 0.12, *p* < 0.01).

The significance of indirect effects of relatedness through plausibility on agreement was calculated using the coefficients that have been presented above and the Prodclin programme, which estimates asymmetric confidence intervals appropriate for indirect effects (see Pituch et al., 2006; MacKinnon et al., 2007). The indirect effect for plausibility was 0.23 (95% confidence interval [0.16; 0.30]) for participants high in need for cognition and 0.09 (95% confidence interval [0.02; 0.15]) for participants low in need for cognition.

In sum, the congruence hypothesis was again supported by the results: participants were more persuaded by arguments involving similar value motives than by arguments involving orthogonal or opposing values. This finding emerged using a design with random pairs of values sampled from around the circular model, analyzed using multilevel modeling. In addition, the multilevel mediational analysis supported the hypothesis that similar values may be more persuasive because their linkage is more plausible and fitting with personal experience. This mechanism was important in participants who were low in need for cognition and among participants high in need for cognition, but the overall impact of relatedness (through plausibility) was stronger among participants higher in need for cognition. Thus, value similarity is particularly important among people who are more likely to carefully scrutinize the arguments, suggesting that this similarity may be an aspect of argument structure that people find useful and rational.

## Experiment 3

A limitation of Experiments 1 and 2 is that both were conducted in a single, Western nation, the United Kingdom. It is well documented that cultures differ in the priority they place on individualistic and collectivist ideologies, which has ramifications for cross-cultural differences between values (Schwartz, 1990; Triandis, 1995). It has been argued that many collectivist cultures also tend to place less emphasis on linear logical thinking, instead embracing a dialectical cognitive framework (Nisbett et al., 2001; Spencer-Rodgers et al., 2010). Notwithstanding these cultural differences, evidence testing the circular model of values has found that relations between values are similar in most nations (Schwartz, 1992, 2006). Thus, from the perspective of the circular model, the effects of co-value arguments should also be similar in different cultures. Experiment 3 tested this implication by attempting to replicate the findings of Experiment 2 in a relatively collectivist nation, India (Suh et al., 2014).

### Method

#### Participants

Participants were 100 undergraduate students (49 women, 51 men) at Karnatak University, who participated for course credit.

#### Procedure

Participants took part in groups from 5 to 18. The procedure was similar to Experiment 2, except that practical constraints prevented us from presenting the materials using a computer. This constraint made it difficult to randomly create the co-value arguments for each participant. Given the robust findings across different sets of co-value arguments in Experiment 2, we instead gave all participants the same set of 20 randomly generated co-value arguments in a single random order. (Value relatedness was calculated in the same way as in Experiment 2.) Below each co-value argument, participants rated their agreement using the same 5-point scale as in Experiment 2. After completing this rating, participants rated the plausibility of the argument using a 3-point scale, from 1 (not at all) to 3 (strongly). After responding to the co-value arguments, participants completed the 18-item Need for Cognition Scale (Cacioppo et al., 1984), as in Experiment 2 (α = 0.66). Participants were then debriefed and thanked for their participation.

### Results and discussion

As in Experiment 2, relatedness, need for cognition (*M* = 2.68, *SD* = 0.739), and plausibility values were recoded to values between 1 and 5. The data were analyzed with the same multilevel models as in Experiment 2. In contrast to Experiment 2, relatedness did not have a main effect on agreement (see Model 1 in Table 3). In Model 2, we added need for cognition and the interaction between relatedness and need for cognition. Need for cognition did not affect agreement, but there was a significant interaction between need for cognition and relatedness. The direction of this interaction was the same as in Experiment 2, and relatedness was positively related to agreement for people scoring one standard deviation above (*B* = 0.09, *p* = 0.03) and one standard deviation below the mean of need for cognition (*B* = 0.02, *p* = 0.58), although the latter simple slope was not statistically significant.

**Table 3:** **Experiment 3: multilevel modeling results (standard errors between brackets)**.

	**Model 1**		**Model 2**		**Model 3**	
**FIXED PARAMETERS**						
Intercept	4.109	(0.065)	4.109	(0.065)	4.105	(0.041)
Relatedness	0.059	(0.039)	0.059	(0.039)	0.016	(0.022)
Need for cognition (NFC)			0.082	(0.052)	0.034	(0.042)
Relatedness*NFC			0.048^**^	(0.018)	0.024	(0.015)
Plausibility					0.462^***^	(0.014)
Plausibility*NFC					0.085^***^	(0.018)
**RANDOM PARAMETERS**						
Participant variance	0.112	(0.021)	0.110	(0.021)	0.072	(0.014)
Value pair variance	0.055	(0.021)	0.055	(0.021)	0.015	(0.007)
Residual variance	0.770	(0.025)	0.768	(0.025)	0.504	(0.016)

*Estimates are unstandardized multilevel regression coefficients. In order to obtain standardized effect sizes, divide the unstandardized coefficients by the square root of the residual variance of Model 1*.

** p < *0.01*;

*** *p < *0.001**.

Model 3 added plausibility to the model. We also added interaction terms between need for cognition and plausibility to the model. Plausibility was positively related to agreement but there was also an interaction between plausibility and need for cognition (see Model 3 in Table 3). The positive relation between plausibility and agreement was stronger for people at one standard deviation above the mean of need for cognition (*B* = 0.52, *p* < 0.001) than for people one standard deviation below the mean of need for cognition (*B* = 0.40, *p* < 0.001). This interaction between plausibility and need for cognition was close to zero in Experiment 2. The weaker effect of plausibility in Experiment 3 compared to Experiment 2 and the interaction with need for cognition could be due to genuine differences between the UK and India in terms of a tendency for less of a linear analytic style of thinking in collectivist cultures (Nisbett et al., 2001; Spencer-Rodgers et al., 2010). However, they could also be related to our use of a 3-point scale for plausibility in Experiment 3 compared to a 5-point scale in Experiment 2.

Regarding mediation, adding plausibility to the model made the interaction between relatedness and need for cognition non-significant. We continued the mediation analysis as in Experiment 2 and analyzed whether relatedness affected plausibility (the mediator). Relatedness had a near-significant effect on plausibility (*B* = 0.09, *p* = 0.051). In contrast to Experiment 2, the effect of relatedness on plausibility was not qualified by an interaction with need for cognition in this analysis (*B* = 0.03, *p* = 0.25).

The significance of indirect effects of relatedness through plausibility on agreement was calculated in the same way as in Experiment 2. The indirect effect for plausibility was 0.05 (95% confidence interval [0.01; 0.11]) for participants high in need for cognition and 0.03 (95% confidence interval [−0.01; 0.07]) for participants low in need for cognition. Thus, the indirect effect was only significant for participants high in need for cognition, which is the same pattern as for the effect of relatedness on agreement.

In sum, Experiment 3's Indian sample replicated the main results from Experiment 2's British sample: participants were more persuaded by arguments involving similar value motives than by arguments involving orthogonal or opposing values, albeit only for participants high in need for cognition. This finding again emerged using a design with random pairs of values sampled from around the circular model, analyzed using multilevel modeling. In addition, the effect of value similarity was again stronger among participants high in need for cognition than among participants low in need for cognition, with argument plausibility playing a mediating role. Thus, value similarity was more important among people who are more likely to carefully scrutinize the arguments, despite the change of culture.

## Experiment 4

Experiment 4 attempted to demonstrate the relevance of our findings for real-world argumentation. Although the simple style of the arguments used in Experiments 1, 2, and 3 are consistent with plentiful examples of co-value argumentation in philosophy, politics, and rhetoric, such arguments are also frequently embedded within other information. This is important when considered in connection with the findings of Experiments 2 and 3, wherein participants who were more likely to enjoy effortful cognitive tasks exhibited stronger effects of value relatedness on their acceptance of the simple co-value arguments. The fact that effortful thought aided the success of the arguments suggests that effects value relatedness should also occur if the co-value arguments are longer and more complex.

To examine this issue, Experiment 4 examined the effects of value relatedness within a realistic piece of political propaganda, embedding the value argument in a real-world persuasion context. To achieve this aim, actual policy statements from a British political party were slightly modified and presented to participants. Participants rated the persuasiveness of the policy statements (arguments) they received and the importance of the reason and target values to them personally. Because we had already shown the value relatedness effect across numerous pairs of social values, participants in this experiment were presented with only one argument, which either involved similar or opposing motives. Our aim was to show that relatedness could have the same effects on plausibility and agreement even when embedded in a longer political argument.

### Method

#### Participants

Participants were 82 undergraduate students at a British university, who were paid £1 for their participation, in a coffee shop.

#### Procedure

Participants completed a questionnaire containing the manipulation of co-value argumentation, followed by measures of argument persuasiveness, argument plausibility, and value importance. Participants were then debriefed and thanked for their participation.

#### Manipulation of co-value argumentation

The manipulation exposed participants to a policy statement that was taken from the political manifesto of a British political party. To avoid the influence of affiliations to a British party, all participants were informed that this policy statement was taken from the (fictional) FDP party in Australia. The policy related to social justice, and we manipulated whether it was supported by a similar social value (broadmindedness) or an opposing social value (wealth). The similar-value policy statement is shown below:

“FDP is the party of social justice and broadmindedness. Reforms introduced by the FDP have built a fairer and more just society. Our country now has the most comprehensive social justice legislation in the United Nations and our commitment to protection for every citizen is also enshrined in the 1998 Human Rights Act.

In addition, we have introduced a range of policies which protect people from discrimination on the grounds of disability, help more disabled people to find and stay in work and support those whose disabilities mean they are unable to work.

We know that legislation alone cannot achieve the systemic and cultural changes we need to make diversity and human rights core values in our society. So, in order to bring about measurable improvement in the position of those who are discriminated against, we believe that all our citizens should receive education that encourages them to be broadminded and tolerant in every aspect of their lives. Increasing broadmindedness *will* increase the fairness of our society.”

The opposing-value policy statement was the same, except that the first sentence became “FDP is the party of social justice and wealth” and the last two sentences were as follows:

“So, in order to bring about measurable improvement in the position of those who are discriminated against, we believe that all our citizens should be able to strive to secure greater wealth through every aspect of their lives. Increasing the pursuit of wealth *will* increase the fairness of our society.”

#### Measures

***Argument persuasiveness***. The argument persuasiveness measures included four questions: “How persuaded were you by this policy statement?,” “How convinced were you by this policy statement?,” “If all other variables were equal, would you vote for this party on the basis of this policy statement?,” and “Aside from how important you consider these issues to be, how strong do you find this policy statement?” Participants answered these questions using 10-point scales from 1 (not at all) to 10 (extremely) or from 1 (not at all) to 10 (definitely) (α = 0.82).

***Argument plausibility***. Participants completed two questions that assessed the plausibility of the argument: “How plausible did you find this policy statement?” and “To what extent do you find this policy statement believable?” Participants answered these questions on 10-point scales from 1 (not at all) to 10 (extremely) (*r* = 0.63).

***Value importance***. Participants rated the importance of the target and reason values (wealth, broadmindedness, and social justice) using the same scale as described in Experiment 1. These were interspersed among nine filler values from around Schwartz's model.

### Results and discussion

#### Argument persuasiveness

A One-Way (value relatedness: similar vs. opposing) ANOVA was conducted on participants' ratings of argument persuasiveness. Again, participants were more persuaded by the political policy statement that cited values serving similar motives (*M* = 5.99) than by the statement using opposing values (*M* = 5.13), *F*_(1, 80)_ = 7.65, *p* < 0.01, *d* = 0.62.

#### Value importance

The correlations between target and reason value importance and persuasiveness and plausibility were non-significant (all *p*s > 0.25). Also, a regression analysis revealed that participants were more persuaded by the policy statement that cited a similar value in support of social justice than by the policy statement that cited an opposing value, even after controlling for the importance of both target and reason values, *b* = 0.94, *t*_(78)_ = 2.96, *p* < 0.005. Therefore, value importance again did not account for the link between value relatedness and argument persuasiveness.

#### Argument plausibility

To investigate our hypothesis that the plausibility of the argument plays a role in the relatedness effect, we tested whether participants found the policy statements to be differentially plausible. A One-Way (value relatedness: similar vs. opposing) ANOVA revealed that there was a significant effect of the experimental manipulation on participants' ratings of argument plausibility, *F*_(1, 80)_ = 11.35, *p* < 0.001, *d* = 0.75. Consistent with the effects on the argument persuasiveness ratings, participants found the policy citing similar values to be more plausible (*M* = 5.83) than the policy citing opposing values (*M* = 4.79).

**Figure 3:**
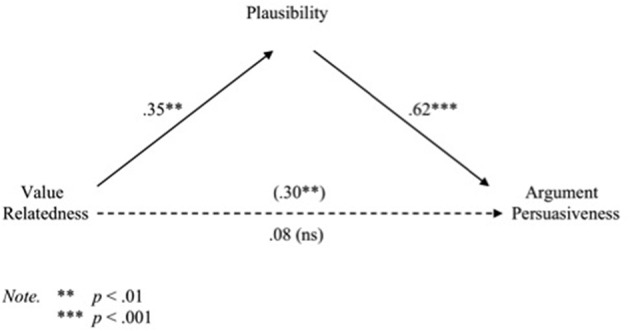
**Path diagram showing the mediational link between value relatedness and argument persuasiveness via plausibility (standardized regression coefficients) in Experiment 4**.

Baron and Kenny's (1986) three-step test of mediation were then applied (see Figure 3). Consistent with the ANOVAs above, regression analyses indicated that value relatedness significantly predicted participants' ratings of argument plausibility (mediator), *b* = 1.04, *t*_(80)_ = 3.37, *p* < 0.001, and that value relatedness significantly predicted participants' argument persuasiveness ratings, *b* = 0.86, *t*_(80)_ = 2.77, *p* < 0.01. In addition, a regression analysis that included value relatedness and argument plausibility as simultaneous predictors of argument persuasiveness revealed a significant effect of argument plausibility on argument persuasiveness, *b* = 0.61, *t*_(80)_ = 6.73, *p* < 0.0001, while the influence of value relatedness was reduced to non-significance, *b* = 0.23, *t*_(79)_ = 0.85, *p* < 0.40. The Sobel test confirmed mediation, *z* = 3.01, *p* < 0.003.

It is also worth noting that our measure of argument persuasiveness included a question relating to behavioral intention: participants' likelihood of voting for the party on the basis of this policy statement. Regression analyses using this measure as the dependent variable revealed an identical pattern of mediation. That is, consistent with the second requirement of mediation (the first requirement was the same as in the above analysis), value relatedness significantly predicted participants' willingness to vote for the party, *b* = 1.15, *t*_(80)_ = 2.55, *p* < 0.02. In addition, argument plausibility significantly predicted participants' voting preference, *b* = 0.60, *t*_(79)_ = 4.50, *p* < 0.001, and reduced the influence of value relatedness to non-significance, *b* = 0.56, *t*_(79)_ = 1.32, *p* < 0.19. The Sobel test also confirmed mediation, *z* = 2.71, *p* < 0.01. Thus, participants who received the argument that used similar values were more willing to vote for this party than participants who received the argument that used opposing values, because they found the similar value arguments more plausible.

## General discussion

Across four experiments, we investigated the impact of the psychological relation between values on the persuasiveness of co-value arguments. Experiment 1 revealed that people are more persuaded by arguments involving similar values than by arguments involving orthogonal or opposing values. Experiment 2 replicated this effect in a multilevel analysis using random pairs of values, while also finding that the effect is increased by deep thought about the arguments (as indexed by individual differences in need for cognition) and mediated by tendencies to see links between compatible values as being more plausible. Experiment 3 extended these conclusions to a sample in a nation with a relatively collectivist ideology. Finally, using real-life persuasive arguments derived from political policy statements, Experiment 4 again found a significant effect of value-relatedness on ratings of argument strength, while providing further support for the hypothesis that the effects of value relatedness on persuasion occur through its influence on the plausibility of the argument.

Together, these results indicate that the motivational compatibility of values plays an important role in co-value argumentation. This research has provided the first direct empirical examination of co-value argumentation and indicates that value relatedness can now be used as an *a priori* predictor of argument persuasiveness. An important implication of this evidence is that, although appealing to diverse values may have the positive outcome of appealing to a more diverse range of voters (Gordon and Miller, 2004), the effectiveness of this strategy may be undermined by a reduction in the persuasiveness of the arguments to the audience. When arguments contained similar values as support for each other, participants in our research perceived the arguments linking the values as more plausible and hence showed more agreement with the arguments. This was true across a wide variety of values, using two-sentence arguments about research findings, and using complex policy statements taken from political party literature.

Of course, our designs possessed some limitations. For instance, we always measured argument agreement before argument plausibility ratings because our principal interest was in the agreement variable. Thus, we wished to obtain untainted agreement ratings. We wanted these to appear first so that participants did not feel as though the agreement had to follow from the plausibility ratings. Nevertheless, it would be useful to have comparison data with the plausibility ratings first.

Also, our designs did not test whether the effects of value relatedness are moderated by the importance of the value that is being promoted. Prior research on attitude function matching in persuasion has found that messages are more persuasive when they promote a value that a person cherishes, rather than an irrelevant concern (DeBono, 2000; Maio and Olson, 2000). These effects appear to rely, in part, on the ability of message matching to elicit greater scrutiny of the message (Petty and Wegener, 1998; Blankenship and Wegener, 2008). Given that the present effects are facilitated by deep thought (Experiments 2 and 3) and are present even for more complex messages (Experiment 4), it is conceivable that the effect of value relatedness is even stronger when the arguments concern values that are considered to be highly important than when the arguments concern values are considered to be less important. Recall, however, that the effects of value relatedness were unattributable to differences in value importance in the two experiments (1 and 4) where we collected data on value importance for the target and reason values. Nonetheless, neither study had sufficient power to detect differences in the effects of value relatedness at different levels of value importance (i.e., the moderation effect). To address this issue robustly, future research will need to consider pre-argument value importance across a variety of values and value pairs. At the same time, however, the research would have to measure prior value importance for both the target value and the supporting value. Such an approach would require additional measures in a design that avoids complications from priming value importance (e.g., by separating measures over time), and multilevel modeling that is more complex than we employed here.

The integration of ideas from recent reasoning and argumentation research within cognitive psychology and research on values in the social domain raises other provocative avenues to explore. For instance, one interesting issue is whether the effects of value relatedness depend on the source of the arguments. Are these effects greater or weaker when people are processing an argument endorsing a value, but from a source that they dislike? For instance, our results indicate that people should be more likely to agree that forgiveness promotes helpfulness (motivationally congruent values) than to agree that forgiveness promotes power (motivationally opposing values). An interesting question is whether such a difference would not emerge when the source is a group or person who is disliked. If the statements come from a disliked political group, for instance, they may elicit less attention and therefore a lower effect of value relatedness. At the same time, we may distrust abstract statements from disliked groups, because we are suspicious of their concrete instantiations of the values at stake. This effect would be ironic, because shared perspectives on values are potential means for bridging gaps between opposing factions, but not if a distrust of the sources leads to less appreciation of the sentiments. This issue is important because there is considerable evidence for diverse effects of argument sources on persuasion, operating through effects on the valence of reactions to message arguments (see Petty and Wegener, 1999) and meta-cognitive confidence in those reactions (e.g., Petty et al., 2007; Tormala et al., 2007; Briñol and Petty, 2009). The role of source characteristics has also drawn the attention of cognitive psychologists concerned with argument strength (e.g., Corner and Hahn, 2009; Hahn et al., 2009; Harris and Hahn, 2009; Harris et al., 2012; Lagnado et al., 2012), giving rise to complementary perspectives that would benefit from closer integration.

It would also be useful to consider the potential of different models of values to predict the acceptability of co-value arguments. Our approach was based on Schwartz's model because of its explicit predictions about motivational conflicts and compatibilities between values. Other important models do not posit such motivational conflicts and compatibilities, but they have distinctions that are also worth investigating. For example, Rokeach (1973) distinguishes between instrumental (ways of acting; e.g., helpfulness) and terminal values (ends states of existence; e.g., freedom), and it is plausible that the instrumental values are more convincing as argumentative antecedents than are terminal values. In addition, Trapnell and Paulhus (2012) distinguish between values dedicated to social communion with others versus values aimed at personal agency and accomplishment, and there is evidence that famous people who use agentic values as means to communal values are more likely to be regarded as moral heroes than famous individuals who pursue agency as its own goal (Frimer et al., 2012). Thus, although our research was not designed to examine these distinctions between values, they may also affect the extent to which co-value arguments are persuasive.

Notwithstanding these issues for future research, the present evidence consistently revealed that it is now possible to predict which arguments will be seen as more compelling, based on their connections to values. This has important ramifications for understanding political attitudes and their connections to values. Returning to the example in the beginning of this paper, it seems that both the 19th Century Communist Party and a former US President fortuitously linked values that are motivationally congruent. People value the consistency and plausibility that such motivational congruence provides, and this may be a major mechanism through which complex assertions about values become compelling or not.

### Conflict of interest statement

The authors declare that the research was conducted in the absence of any commercial or financial relationships that could be construed as a potential conflict of interest.
